# Correction: Electrocatalytic hydrogen generation using tripod containing pyrazolylborate-based copper(ii), nickel(ii), and iron(iii) complexes loaded on a glassy carbon electrode

**DOI:** 10.1039/d2ra90027k

**Published:** 2022-04-22

**Authors:** Mohamed M. Ibrahim, G. A. M. Mersal, Ahmed M. Fallatah, Khaled Althubeiti, Hamdy S. El-Sheshtawy, Manal F. Abou Taleb, Manash R. Das, Rabah Boukherroub, Mohamed S. Attia, Mohammed A. Amin

**Affiliations:** Department of Chemistry, College of Science, Taif University P.O. Box 11099 Taif 21944 Saudi Arabia Ibrahim@tu.edu.sa mohamed@tu.edu.sa; Chemistry Department, Faculty of Science, Kafrelsheikh University Kafr El Sheikh 33516 Egypt; Department of Chemistry, College of Science and Humanities in Al-Kharj, Prince Sattam Bin Abdulaziz University Al-Kharj Saudi Arabia; Polymer Chemistry Department, National Center for Radiation Research and Technology (NCRRT), Egyptian Atomic Energy Authority Cairo Egypt; Advanced Materials Group, Materials Sciences and Technology Division, CSIR-North East Institute of Science and Technology Jorhat 785006 Assam India; Academy of Scientific and Innovative Research (AcSIR) Ghaziabad 201002 India; Univ. Lille, CNRS, Centrale Lille, Univ. Polytechnique Hauts-de-France, UMR 8520 – IEMN F59000 Lille France; Chemistry Department, Faculty of Science, Ain Shams University Abbassia 11566 Cairo Egypt Mohd_mostafa@sci.asu.edu.eg

## Abstract

Correction for ‘Electrocatalytic hydrogen generation using tripod containing pyrazolylborate-based copper(ii), nickel(ii), and iron(iii) complexes loaded on a glassy carbon electrode’ by Mohamed M. Ibrahim *et al.*, *RSC Adv.*, 2022, **12**, 8030–8042, https://doi.org/10.1039/D1RA08530A.

The authors regret that an incorrect version of Scheme 1 was given in the original article. The corrected version is shown here.

**Scheme 1:**
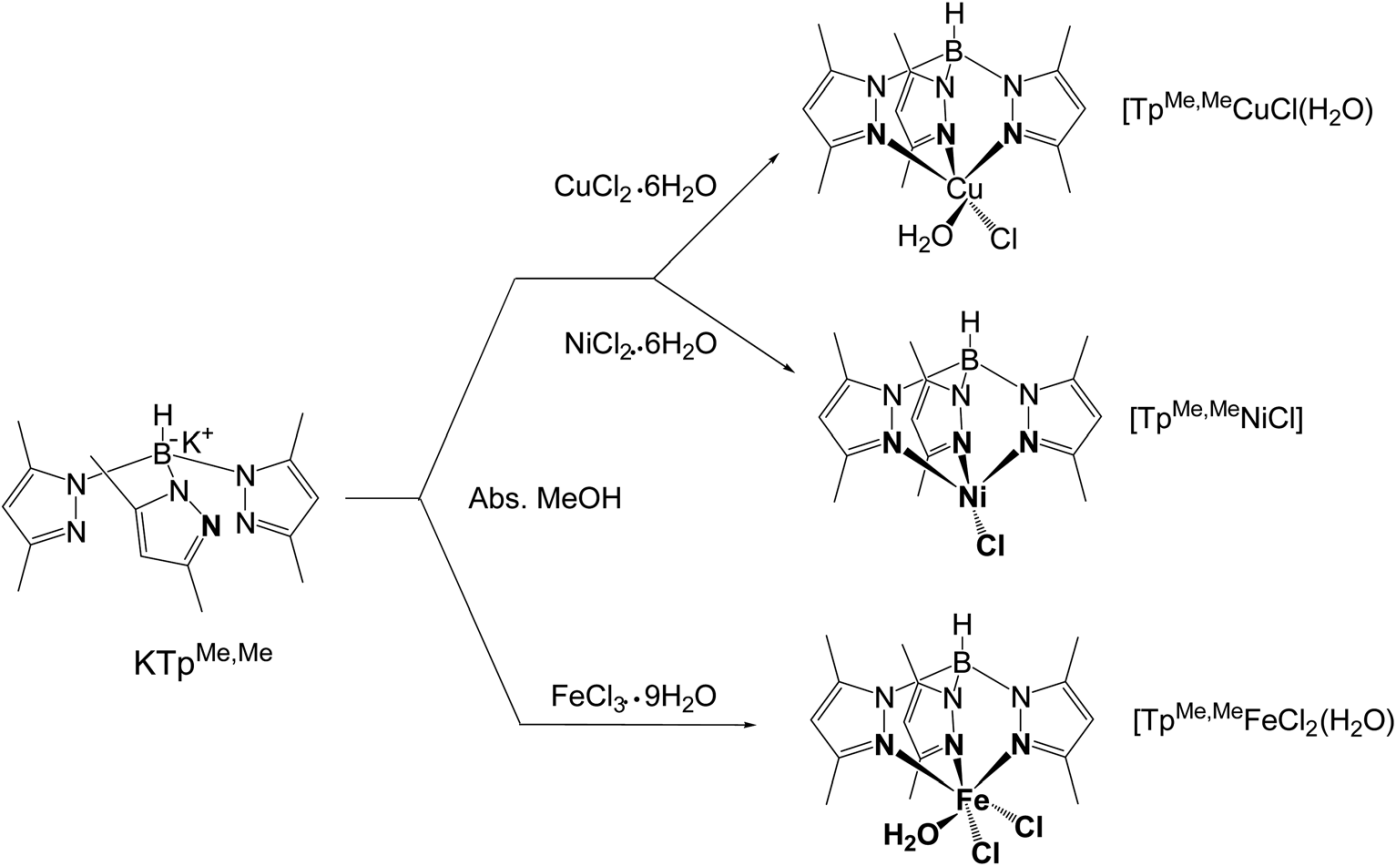
The structures of ligand KTp^MeMe^ and its metal complexes MC.

The Royal Society of Chemistry apologises for these errors and any consequent inconvenience to authors and readers.

## Supplementary Material

